# Postbiotics as Emerging Strategy Targeting Obesity- and Aging-Related Breast Cancer—Prospects in Prophylaxis and Therapy

**DOI:** 10.3390/life16040628

**Published:** 2026-04-08

**Authors:** Joanna Wasiak, Katarzyna Anna Oszajca, Janusz Szemraj, Monika Witusik-Perkowska

**Affiliations:** 1 Department of Medical Biochemistry, Medical University of Lodz, 6/8 Mazowiecka Str., 92-215 Lodz, Poland; joanna.wasiak@umed.lodz.pl (J.W.); janusz.szemraj@umed.lodz.pl (J.S.); monika.witusik-perkowska@umed.lodz.pl (M.W.-P.); 2 International Doctoral School, Medical University of Lodz, 90-419 Lodz, Poland

**Keywords:** breast cancer, postbiotics, microbiome, aging, obesity, estrogens, oxidative stress, inflammation, cellular senescence, oncotherapy

## Abstract

Aging and obesity accompanied with hormonal disequilibrium represent critical, inter-related risk factors for breast cancer, significantly influencing disease incidence, progression, and therapeutic outcomes. This review aims to elucidate the multifaceted biological mechanisms linking obesity and aging to breast carcinogenesis, with a particular focus on the emerging therapeutic and preventive potential of postbiotics as molecules targeting cellular events important for cancer growth and responsiveness. Despite continuous advancement, breast cancer therapy still poses several challenges, such as treatment-induced acquired resistance, which is boosted by the inflammatory phenotype of senescent cancerous cells, as well as undesired side effects resulting from the destruction of normal cells. Such a complex background of breast carcinogenesis and oncotherapy resistance opens avenues to search for new preventive approaches and adjunctive treatment regimens. Postbiotics demonstrate a variety of benefits due to their selective antineoplastic activity, as well as the cytoprotective potential associated with antioxidant, anti-inflammatory and anti-senescent properties. Pleiotropic effects of postbiotics make them a promising tool for counteracting cellular and physiological disturbances that favor breast cancer development, including age- and obesity-related factors. They are prospective adjunctive agents in oncotherapy, albeit their efficacy and safety need to be thoroughly evaluated in clinical studies prior to implementation in routine treatment modes.

## 1. Introduction

Breast cancer is one of the most common cancers among females and is associated with high mortality and morbidity rates. The major risk factors include environmental influences such as age and hormonal disequilibrium, obesity, sedentary lifestyle, and alcohol consumption, as well as genetic predispositions, particularly *BRCA1* and *BRCA2* mutations. The development of breast cancer is thought to be linked to an imbalance in the management of sex hormones, particularly estrogen exposure. Therefore, changes in hormonal balance throughout life, with menopause as a turning point, are important factor influencing the pathogenesis of breast cancer [1].

Although breast carcinogenesis is driven by an interplay of genetic, environmental and lifestyle variables, the increasing incidence of breast cancer in countries that have adopted Western habits and diets suggests that exogenous factors play a significant role in cancer risk development. Conversely, the decline in mortality rates in regions with access to advanced prevention and early intervention emphasizes the importance of prophylaxis in minimizing cancer harvest [2]. As age and obesity are positioned within the major causes of breast cancer, efforts to promote a healthy lifestyle throughout life, as well as to counteract the metabolic consequences of accelerated aging and obesity, should be treated as serious scientific challenges. A growing number of reports provide strong evidence that healthy lifestyle and dietary composition can target molecular and cellular events that prevent cells from transitioning to a cancerous state. Dietary products or supplements containing bioactive compounds with antioxidant and anti-inflammatory properties are particularly promising health promoters.

Breast cancer is classified according to both its morphological features and its molecular profile. Molecular subtyping—determined by hormone receptor status and the expression of human epidermal growth factor receptor 2 (HER2)—has become essential for guiding treatment decisions and predicting patient outcomes. Advances in molecular techniques have enabled researchers to categorize breast cancer into several subtypes, including luminal A, luminal B, HER2-positive, and triple-negative breast cancer (TNBC). Luminal A tumors are characterized by the presence of hormone receptors, specifically estrogen receptors (ER) and progesterone receptors (PR), and are therefore commonly referred to as ER-positive cancers. Luminal B tumors also express ER and may express PR, but they often show higher proliferative activity and may additionally express HER2. HER2-positive breast cancer is defined by the overexpression of HER2 receptors and typically lacks hormone receptor expression. In contrast, TNBC does not express ER, PR, or HER2, and it is generally considered the most aggressive subtype [3]. Approximately 70–80% of breast cancer cases are ER-positive. Although treatment response depends on the stage of the disease, the median overall survival for this subtype is approximately five years. The standard approach to breast cancer treatment includes surgery, chemotherapy, and radiation therapy. Surgical management may involve mastectomy or breast-conserving surgery and is frequently complemented by adjuvant treatments such as radiotherapy, chemotherapy, and hormone therapy [4].

Unfortunately, standard chemotherapy is often associated with significant adverse effects, including nausea and vomiting, cardiotoxicity, hepatic and renal dysfunction. In addition, breast cancer-specific mechanisms of immune evasion frequently contribute to disease progression and resistance to immunotherapy [5]. Treatment resistance in breast cancer arises through multiple adaptive mechanisms. Cancer cells often activate pro-survival signaling pathways, such as PI3K/AKT/mTOR and MAPK, which promote proliferation and inhibit apoptosis despite therapeutic pressure. In addition, epigenetic alterations—including DNA methylation, histone modifications, and changes in non-coding RNA expression—can modify gene expression patterns associated with drug sensitivity for cisplatin and doxorubicin. Remodeling of the tumor microenvironment further contributes to resistance by enhancing interactions between cancer cells, stromal cells, and immune components, leading to increased inflammation, immune evasion, and protection from anticancer therapies. Together, these processes enable tumor cells to survive treatment and drive disease progression [6].

Moreover, the development of drug resistance significantly limits treatment outcomes. Therefore, there is a crucial need to find novel therapeutic strategies that can improve treatment efficacy while enhancing patients’ quality of life. Recent advancements have shifted breast cancer management towards precision oncology, involving treatment regimens based on diagnoses supported by nuanced molecular subtyping and the development of targeted systemic therapies.

Alongside the expansion of personalized treatment options, critical advancements have led to the identification of new cancer-resistance mechanisms, such as the effect of tumor-associated microbiota, the impact of the circadian rhythm on the spread of metastases and the side effects of treatment within neoplastic cells and the tumor microenvironment, which can paradoxically lead to cancer relapse. Such findings open the avenue for exploring new therapeutic possibilities [2]. Apart from efforts focusing mainly on the development of new chemotherapeutic options, investigations on supportive treatment strategies are also worthy of highlighting. The implementation of certain adjunctive compounds can boost the efficacy of standard treatment regimens and mitigate side effects. Recent studies have emphasised the promising potential of bioactive compounds of natural origin, including postbiotics, which are metabolites or byproducts of probiotic bacteria that benefit health outcomes, although there is currently no clinical evidence that their effectiveness may vary depending on the subtype of breast cancer.

## 2. Aging and Obesity as Breast Cancer Risk Factors

In women, aging and obesity represent two of the most significant and interconnected risk factors that escalate the incidence and progression of breast cancer. While distinct in their biological origins, these factors share a complex pathological synergy mediated by a network of shared molecular pathways [7,8]. Central to this intersection is chronic low-grade inflammation and elevated oxidative stress, which exacerbate cellular damage and impair DNA repair mechanisms [9]. These conditions are further intensified by adipokine signaling imbalances—notably a shift toward pro-tumorigenic leptin levels—and systemic insulin resistance, which promotes cell proliferation via the IGF-1 axis. Furthermore, both aging and obesity drive hormonal dysregulation, primarily by increasing local estrogen production in mammary adipose tissue [10]. These systemic disturbances are increasingly linked to dysbiosis in systemic and mammary microbiotas, where a loss of microbial diversity and the expansion of pro-inflammatory species further disrupts hormonal homeostasis and genomic stability [11,12]. Collectively, these four pillars—inflammation, adipokine signaling, insulin resistance, and hormonal imbalance—foster a mutagenic landscape permissive of malignancy. The following subsections detail the individual and synergistic roles of biological aging and adiposity in the context of breast cancer pathophysiology.

### 2.1. Breast Cancer and Aging

During the process of aging, breast tissue undergoes various morphological, cellular and molecular events which can favor breast diseases, including cancer. Importantly, breast tissue exhibits accelerated aging compared to other organs, and age is considered as one of the most important risk factors for breast cancer development, confirmed by the fact that more than 80% of breast cancer incidents occur after the age of 50 [13,14]. Changes in the structure and function of the breast depend on hormonal balance, including the impact of estrogen. Thus, menopause is considered a stage of life that influences the risk of developing breast cancer and the mechanisms underlying carcinogenesis.

Recent studies suggest a potential association between gut dysbiosis and an increased risk of breast cancer, as alterations in the gut microbial composition may affect estrogen metabolism and related signaling pathways [15]. Especially during menopause, significant hormonal and epithelial alterations occur. This stage of the female life cycle is distinguished by changes in circulating estrogens and other sex hormones. The presence or lack of estrogen appears to modulate gut microbiota homeostasis and age-related disorders in women. Shifts in microbiota composition may have a crucial influence on the development or progression of osteoporosis, weight gain and lipid deposition or breast cancer [16]. One of the potential mechanisms linking gut microbiota to estrogen-dependent carcinogenesis involves the microbial regulation of estrogen metabolism. The gastrointestinal microbiome is recognized as a major determinant of systemic estrogen levels through a functional gene pool referred to as the estrobolome. This term refers to the collective bacterial genes whose enzymatic products, including β-glucuronidase and β-glucosidase, catalyze the deconjugation of estrogen metabolites. Under normal physiological conditions, free estrogens undergo hepatic conjugation followed by excretion in urine or feces. However, a substantial fraction of these conjugated estrogens is hydrolyzed by gut microbial enzymes before elimination, allowing the estrogens to be reactivated and reabsorbed into circulation. This enterohepatic recycling contributes to the increased availability of biologically active estrogens, which may consequently stimulate estrogen receptor signaling within breast tissue. Such estrogen-driven proliferation has been implicated in the initiation and development of breast cancer, particularly ER-positive tumors in postmenopausal women. Disruption of the gut microbial community (dysbiosis) can therefore lead to imbalances in β-glucuronidase activity and altered estrogen homeostasis. Elevated systemic estrogen levels represent a well-established risk factor for hormone-dependent malignancies, including breast cancer [17,18].

On the other hand, the age-related decline in estrogen production during menopause has been linked to an increased risk of metabolic diseases and premature death, since estrogens preserve energy homeostasis and promote metabolic health. Although it remains unclear whether reduced estrogen signaling during menopause is a major cause of age-related changes to homeostasis, it is undeniable that a decrease in estrogen accelerates aging [19]. Despite the data being not fully consistent, recent reports have suggested that estrogens can prevent cellular events that favor aging, such as DNA damage, telomere shortening, oxidative stress, cellular senescence, and an inflammatory phenotype [20,21]. The in vitro findings are supported by reports showing a decline in markers of inflammation and senescence resulting from menopausal hormone therapy [22,23,24].

The aging process is accompanied by a gradual increase in the number of senescent cells found in all tissues. Cellular senescence induced by internal or external stimuli could be considered as a complex stress response that involves molecular and phenotypic changes. It resulted in irreversible replicative arrest, caused by the upregulation of cell cycle inhibitors such as p16INK4A and/or p21CIP1, apoptosis resistance, metabolic dysfunction, overproduction of reactive oxygen species (ROS), and gaining of a senescence-associated secretory phenotype (SASP) [25]. Senescent cells may operate in an autocrine or paracrine manner through SASP; however, they exert a negative impact not only on their close neighborhood, but also on distant tissues; thus, the accumulation of them can cause significant organism dysfunction via a mechanism resembling a chain reaction or snowball effect. The SASP factors can include pro-inflammatory cytokines, such as tumor necrosis factor-α (TNF-α), viral FK506-binding protein (vFKBP), interleukin IL-6 and IL-8, chemokines, and extracellular matrix proteases, as well as bioactive lipids, non-coding RNAs and extracellular vesicles. SASP is also considered pro-tumorigenic, since it promotes a spectrum of events facilitating the invasion and metastasis of neoplastic cells, while senescent cancer cells present the ability to generate more aggressive progeny, being a consequence of their abnormal division or reprogramming [26,27].

Cell senescence in healthy tissues is partially regulated by wild-type p53. Since *TP53* gene mutations are common in cancer cells, the uncontrolled spread of the cell senescence process within the tumor microenvironment may exacerbate the chronic inflammation induced by the SASP. SASP has been shown to promote tumor development, progression, and metastasis of neoplastic cells of various origins, including breast cancer. SASP secretome consists of several factors favoring tumor expansion, such as growth factors, matrix metalloproteinases, chemokines and pro-inflammatory cytokines. IL-6 and IL-8, being the dominant cytokines of SASP, can induce epithelial–mesenchymal transition (EMT) and promote epithelial migration and invasion, stimulate angiogenesis and tumor growth, disrupt cell–cell communication, alter macrophage function, and trigger innate immune response [28].

At the level of cellular machinery, aging of the mammary gland is accompanied by changes in several molecular signaling pathways that regulate cellular senescence, proliferation, and differentiation. These signaling alterations contribute not only to functional decline of the tissue but also could increase susceptibility to breast cancer. A pro-carcinogenic microenvironment is a consequence of inter-related molecular events triggered by the accumulation of senescent epithelial and stromal cells that secrete SASP factors (e.g., IL-6, IL-8, MMPs, chemokines). The senescence-altered microenvironment promotes chronic inflammation driven by STAT3 and NF-κB activation. Specifically, chronic DNA damage and oxidative stress in senescent cells activate the IKK complex, leading to NF-κB nuclear translocation and SASP secretion. A key component of SASP, IL-6, acts in an autocrine manner to activate Janus kinases (JAK), which in turn mediate the phosphorylation and dimerization of STAT3 [28].

Constant autocrine/paracrine STAT3 activation may enhance the expression of genes promoting EMT, stemness and migration, thereby supporting the growth and metastasis of breast cancer cells. Overactivation of the RANK/RANKL pathway in postmenopausal women being a consequence of endocrine shift may cause a dual effect contributing to carcinogenesis. It concurrently promotes epithelial proliferation via NF-κB and cyclin D1 activation and induction of epithelial senescence through p16/p19-dependent pathway, paradoxically pushing cells toward both hyperplasia and senescence. RANKL-rich microenvironments in older breast tissue may predispose tumor formation, supporting initiation and invasiveness of hormone-driven breast cancer. In older women high cyclin D1 expression is linked to ER-positive breast cancer, since in aging breast tissue, upstream age-related growth factors upregulate cyclin D1 promoting abnormal epithelial cell proliferation. Aging can also alter the activity of Slug/Snail pathway regulating DNA damage response and senescence in mammary epithelial cells. Slug downregulation impairs the mechanism of DNA damage response (DDR) causing accelerated accumulation of DNA damage and premature epithelial senescence. Dysregulation of this pathway may also shift epithelium toward a pro-tumorigenic phenotype with increased genomic instability [25]. One of the canonical age-related pathways is the insulin and insulin-like growth factor-1 signaling (IIS) cascade, playing a central role in the dysregulation of nutrient sensing. IIS activates the PI3K/AKT/mTOR axis and its downstream targets, such as cyclin B, GADD45, 4E-BP1 and S6K, thus impacting the cell cycle, metabolism, apoptosis and stress response. Reduced IIS activity is a characteristic feature of aging that participates in the degradation of terminal duct lobular units (TDLUs) in the mammary gland, which can induce the development of breast pathologies, including cancer [13].

Age-dependent changes in hormonal balance influencing mammary glands may also contribute to the development and progression of breast cancer via pathways involving histone methylation. Epigenetic modifications contribute to genomic stability, DNA repair, and chromatin dynamics, all of which influence breast cancer initiation and progression. Histone/DNA methylation is considered a hallmark of aging, while the methylation status of specific genes also serves as a valuable prognostic indicator for breast cancer patients [29,30]. On the other hand, therapeutic approaches designed to counteract accelerated DNA methylation are regarded as a way to combat age-related diseases, including cancer [31,32]. The disequilibrium in signaling cascades may ultimately shift the mammary tissue from a normal aging state into a pro-tumorigenic, pro-inflammatory, senescence-altered microenvironment favoring breast malignancy [13].

Oxidative stress and inflammation are recognized as key factors in triggering and promoting cellular senescence, a phenomenon that increases gradually with age and is commonly termed ‘oxy-inflammaging’ [33,34]. In postmenopausal women, especially with obesity, the elevated levels of pro-inflammatory cytokines, like IL-1, IL-6, and TNF-α, have been detected. Chronic inflammation promotes oxidative stress, which may create a favorable microenvironment for carcinogenesis. On the other hand, breast cancer patients presented elevated levels of pro-inflammatory cytokines, e.g., IL-6, markers of oxidative stress such as ROS, malondialdehyde (MDA), protein carbonyls (PCC), advanced oxidation protein products (AOPP) and lower levels of antioxidant markers (thiols and ferric-reducing ability of plasma, FRAP) [35]. Such disequilibrium of inflammatory and redox homeostasis has been observed also in postmenopausal women with estrogen-dependent breast cancer [33,36]. Although the majority of published reports recognized oxidative stress and inflammation as factors correlated with carcinogenesis, the data regarding this issue in the context of breast cancer development are confusing, since part of them show no interrelation or even an opposite effect [37,38,39]. Notably, advanced age and menopausal status seem to be important factors modifying the involvement of oxidative stress and inflammation in cancer pathogenesis and progression [40]. While controversy persists with regard to oxidative stress involvement in breast cancer pathogenesis, there are several studies postulating the supportive role of exogenous antioxidants, such as vitamins A, E, and C or melatonin in cancer prophylaxis and treatment. However, patient stratification according to oxidative stress status and breast tumor subtype should be undertaken prior to the implementation of antioxidant-based adjunctive therapy [41,42,43].

Apart from the inherent status of inflammation and the redox state of cancer patients, oncotherapy has also been recognized as a trigger of cellular senescence [44]. Treatment-induced senescence (TIS) may favor cancer therapy escape and subsequent recurrence, as well as destruction of the tumor microenvironment, including healthy cells [45,46]. It has been shown that TIS-related SASP supports treatment escape by inducing EMT and stimulating quiescent tumor cells to re-enter the cell cycle, thereby promoting proliferation and tumor relapse [28]. Senescent cells appeared to be more resistant to apoptosis, thanks to the implementation of self-preventive mechanisms, such as overactivation of anti-apoptotic signaling (e.g., Bcl-2 family members or p53-p21-serpin and phosphoinositide 3-kinase (PI3K)/AKT pathways) [47]. There is also evidence suggesting the occurrence of post-senescence stemness reprograming of cancer cells promoted by SASP. Although the mechanisms of this phenomenon are still under investigation, there are possibilities that phenotypic plasticity of cancer cells allowed for reversal to CSC (Cancer Stem Cell) status by the upregulation of stemness genes (e.g., *CD44*, *CD133*, *OCT4*, *NANOG*) or abnormal division of polyploid giant cells yielding stem progeny [44,45].

This undesired phenomenon was also identified in the case of breast cancer treatment regimens, including radio- and chemotherapy [48,49,50,51]. The development of acquired resistance accounts for up to 50% of patients with breast cancer. Ionizing radiation (IR) has been shown to induce cellular senescence, diminishing the effectiveness of breast cancer treatment, and possibly contributing to late toxic effects [52,53,54]. A plethora of pharmaceuticals have been demonstrated to induce cellular senescence in neoplastic cells, including agents routinely utilized in breast cancer therapy, e.g., doxorubicin, etoposide, cisplatin, and tamoxifen. TIS can be induced via a number of different pathways, with DNA damage and oxidative stress as the main promoters of senescent events [55,56]. To alleviate detrimental effects of inherent senescence of tumor cells or TIS, two adjuvant strategies involving senotherapeutics have been considered [57]. Senomorphics are targeted to redirect the cell machinery to the apoptotic pathway, while senolytics present the selective ability to eliminate senescent cells. Up to date, several compounds have been recognized as potential senotherapeutics, including natural bioactive agents such as quercetin, fisetin, luteolin, or curcumin, with some of them (trabectedin, apigenin, ginsenoside, allicin) also tested in breast cancer studies [54].

### 2.2. Breast Cancer and Obesity

Obesity is thought to be associated with a substantial increase in the risk of breast cancer, particularly in postmenopausal women and with metabolic comorbidities [58]. The underlying mechanisms are complex and involve a variety of hormonal, inflammatory and metabolic factors.

Excessive body weight causes pathological expansion of white adipose tissue (adiposopathy), resulting in hypertrophy and/or hyperplasia of adipocytes, hypoxia, and oxidative stress, accompanied with cellular senescence. This impairs protein secretory pathways and leads to metabolic, inflammatory, immunological and epigenetic changes that favor neoplastic transformation and tumor growth [59,60]. As an endocrine tissue, adipose tissue controls the production and bioavailability of sex hormones (estrogens and androgens), which are regarded as mediators in the association between obesity and breast cancer risk. Increased body fat is associated with higher activity of the aromatase enzyme (encoded by the *CYP19A1* gene), which converts androgens into estrogens and less-active hormones (androstenedione, estrone) into more-potent hormonal forms (testosterone, estradiol) [61]. In postmenopausal women, adipose tissue becomes the main source of estrogen, and the rate of conversion of androgens to estrogens is significantly higher in obese individuals. This process is driven by the upregulation of *CYP19A1* expression in adipose tissue stromal cells, triggered by chronic low-grade inflammation. Specifically, the increased production of pro-inflammatory cytokines, such as TNF-α, IL-6, and prostaglandin E2 (PGE2), acts as a potent stimulator of aromatase activity, leading to significantly higher local estrogen concentrations. Furthermore, aromatase expression is upregulated by leptin, produced by adipocytes in amounts proportional to adipose tissue content; in obese postmenopausal women, this creates a feedback loop that further increases local estrogen production [10,62]. Clinical evidence supports these mechanisms, demonstrating that both the expression of aromatase mRNA and its enzymatic activity are markedly elevated in the breast tissue of overweight and obese postmenopausal women compared to lean individuals [63]. The resulting high levels of estrogens stimulate cell proliferation through their receptors, mainly ERα. The estradiol–receptor complex activates pro-proliferative genes such as *c-Myc*, *Cyclin D1*, and *c-Fos*, driving the G1/S transition of the cell cycle [64]. This enhanced proliferation increases the risk of genetic mutations, facilitating the initiation and development of hormone-dependent breast cancer. In addition, estrogen metabolites may exert genotoxic effects, further elevating the risk of malignant transformation of breast epithelial cells [65].

White adipose tissue is a metabolically active secretory organ that produces a wide range of functional adipokines that regulate numerous physiological and pathological pathways, including insulin sensitivity, glucose and lipid metabolism, appetite, inflammation, immune function, hematopoiesis, and angiogenesis [66,67]. To date, more than ten adipokines have been linked to breast cancer, with the number growing steadily. These include leptin, resistin, visfatin, osteopontin, apelin, chimerin, oncostatin M, and lipocalin, for which a positive correlation between their elevated concentrations and the risk of breast cancer has been observed. Whereas, on the contrary, it has been shown that reduced levels of adiponectin and irisin play a protective role against this disease [68].

Leptin is the main adipokine secreted by adipose tissue that plays a crucial role in regulating appetite and long-term energy balance [66]. In addition to upregulating aromatase activity, leptin acts through its specific receptor (LEPR), which is widely expressed in breast cancer cells, favoring tumor growth by activating signaling pathways, such as JAK/STAT, PI3K/AKT, and MAPK/ERK. These pathways further drive cancer cell proliferation, angiogenesis, and invasion, while inhibiting apoptosis [69]. This adipokine also interacts with the ERα receptor, boosting estrogen signaling pathways, which may accelerate the development of ER-positive breast cancer [70]. Unlike leptin, adiponectin levels are usually reduced in obese individuals. Low levels of adiponectin are associated with increased risk of breast cancer, while higher levels may have a protective effect. Adiponectin inhibits breast cancer cell proliferation by activating AdipoR1 and AdipoR2 receptors on the surface of cancer cells, leading to the induction of AMPK and PPAR-α signaling pathways, which results in cell proliferation inhibition, apoptosis induction, as well as reduced tumor cell migration and invasion. In addition, activation of the AMPK pathway increases glucose uptake in muscles, inhibits glucose production in the liver, and increases fatty acid oxidation, which improves tissue sensitivity to insulin. Thus, adiponectin deficiency promotes the development of insulin resistance, which alters cellular metabolism, creating conditions that support carcinogenesis [68,71]. Moreover, adiponectin has anti-inflammatory properties, and its reduced levels observed in obesity cause increased production of pro-inflammatory cytokines, such as TNF-α and IL-6, in adipose tissue and other organs. Elevated production of pro-inflammatory cytokines can then further suppress adiponectin release, creating a vicious cycle that promotes chronic, low-grade inflammation [72]. Additionally, TNF-α and IL-6 exacerbate insulin resistance by activating NF-κB and JNK signaling pathways, which disrupt insulin signaling at the level of receptors and downstream proteins, maintaining chronic inflammation and metabolic dysfunction [73]. By activating the nuclear factor NF-κB, TNF-α also induces the transcription of genes responsible for the production of growth factors, cytokines, and extracellular matrix degradation enzymes (e.g., MMPs). As a result, cancer cell proliferation, resistance to apoptosis, and migration increase, which promotes invasion and metastasis [74,75]. Moreover, NF-κB regulates the expression of vascular endothelial growth factor (VEGF), which is essential in the process of angiogenesis, enabling the formation of new vessels that nourish the tumor [76]. IL-6 activates the JAK/STAT3 cascade, causing STAT3 to act as an oncoprotein that regulates expression of genes promoting proliferation, apoptosis inhibition, migration, and immunosuppression of the tumor microenvironment [77]. In general, adipokine-induced inflammation affects the breast tissue microenvironment by promoting cell proliferation, disrupting the normal mechanism of apoptosis, and stimulating angiogenesis. High levels of pro-inflammatory cytokines contribute to DNA damage and alter immune cell function, which promotes the escape of cancer cells from immune control.

As adipocytes undergo hypertrophy, some of them exceed their blood supply capacity, leading to hypoxia and the activation of hypoxia-inducible factor 1 (HIF-1). HIF-1 upregulates leptin and VEGF and downregulates adiponectin, which may promote tumor invasion and metastasis [78]. Furthermore, adipocytes that are dead or dying as a result of hypoxia are surrounded by macrophages, forming pathological structures resembling crowns called crown-like structures (CLSs). CLSs are more common in obese women and correlate with metabolic disturbances (insulin resistance, elevated glucose levels and pro-inflammatory cytokines). Macrophages associated with CLS intensively produce pro-inflammatory mediators and are a characteristic marker of white adipose tissue inflammation [79]. Local cytokine and chemokine released by CLS promotes the recruitment of further inflammatory cells and stimulates aromatase expression, increasing local estrogen levels in breast tissue, which contributes to cancer proliferation [80].

Obesity is very often accompanied by insulin resistance, hyperinsulinemia, and hyperglycemia, which are considered to be factors contributing to the development of malignancy. Insulin increases the bioactivity of insulin-like growth factor 1 (IGF-1), which has mitogenic and anti-apoptotic effects, by enhancing its synthesis and reducing the production of insulin-like growth factor-binding proteins 1 (IGFBP-1) and 2 (IGFBP-2) in the liver [81]. High levels of insulin and IGF-1 drive strong activation of both insulin and IGF-1 receptors on breast epithelial cells. This stimulation triggers signaling pathways that promote cell proliferation, inhibit apoptosis, and increase the survival of cancer cells [82,83]. In addition, insulin affects leptin levels by both stimulating its synthesis in adipose tissue as well as preventing its degradation, which leads to elevated leptin concentrations [84]. Conversely, hyperinsulinemia lowers the level of adiponectin, a hormone with anti-proliferative effects. Insulin and IGF-1 also reduce the synthesis of sex hormone-binding globulin (SHBG), resulting in higher estrogen availability and thus increases the risk of hormone-dependent breast cancer [85].

Another factor linking obesity with increased breast cancer risk is oxidative stress. In obese individuals, dysfunctional adipocytes generate excess oxygen free radicals and reactive nitrogen species. This chronic oxidative stress damages cellular structures, including DNA, cell membrane lipids, and proteins, leading to mutations and genomic instability. Oxidative DNA damage, such as changes in nitrogenous bases, DNA strand breaks, and adduct formation, increases the risk of malignant transformation of breast epithelial cells [86]. Prolonged oxidative stress, combined with inflammation, leads to epigenetic changes, including DNA methylation, histone modifications, and regulation by microRNAs. These changes can silence tumor suppressor genes or activate oncogenes, contributing to genome destabilization and promoting carcinogenesis [87]. In addition, pro-inflammatory cytokines TNF-α and IL-6 stimulate immune cells, such as macrophages and monocytes, to increase the production of reactive oxygen and nitrogen species, further escalating oxidative stress. TNF-α and IL-6 can also induce changes in signaling pathways that reduce the activity of antioxidant transcription factors such as Nrf2, decreasing the ability of cells to neutralize free radicals [88].

Obesity also alters gut and breast microbial diversity and relative abundance, which contribute to an elevated risk of breast cancer development and progression by increasing inflammation, causing hormonal disturbances, and weakening immune control [11]. Certain subsets of gut bacteria possess β-glucuronidase enzymes that change the metabolism and enterohepatic circulation of estrogens, increasing their availability in the blood and tissues, which may enhance the proliferation of breast epithelial cells [89]. Dysbiosis leads to a weakening of the intestinal barrier, which increases the permeability of the intestinal walls and the penetration of endotoxins, such as lipopolysaccharides (LPS), into the bloodstream, causing systemic chronic inflammation. Microbial imbalance is also accompanied by reduced production of beneficial microbiome metabolites, such as short-chain fatty acids (SCFA), which under normal conditions preserve leaky gut syndrome, modulate the immune response, and inhibit inflammatory processes, preventing neoplastic transformation [90].

The holistic approach to breast cancer pathogenesis in the context of two major risk factors—aging and obesity—sheds light on the significance of oxidative stress, inflammatory status and cell senescence, not only as important contributors of carcinogenesis but also as the potential targets to adjunctive therapeutic approaches.

## 3. Postbiotic Biology and Bioactivity

**Figure 1:**
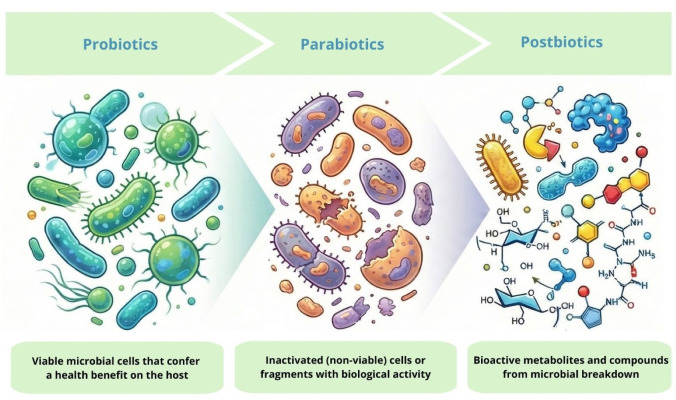
A graphical comparison between probiotics, parabiotics, and postbiotics.

Postbiotics are defined as the bioactive compounds produced by probiotic microorganisms during fermentation or released after microbial cell lysis. Among these, lactic acid bacteria (LAB), particularly *Lactobacillus* and *Bifidobacterium* species, are the most extensively studied producers of postbiotic substances. Apart from postbiotics obtained from microbial cultures, they occur naturally in a variety of fermented foods, such as fermented dairy products (yogurt, kefir), kombucha or pickled vegetables [91]. For clarity, probiotics are live microorganisms that confer health benefits, parabiotics (inactivated probiotics) are defined as non-viable microbial cells and postbiotics are metabolites or byproducts derived from these microorganisms (Figure 1).

Postbiotics can be classified based on their chemical composition and include lipids (e.g., short-chain fatty acids), carbohydrates (e.g., teichoic acids), enzymes, vitamins, peptides (e.g., bacteriocins), and organic acids [30,92]. In addition to soluble metabolites and macromolecules, probiotic bacteria—such as *Lactobacillus* species—can produce extracellular vesicles (EVs) which have recently been recognized as a novel class of postbiotics. These EVs facilitate intercellular communication among microbial populations and influence numerous biological processes, including cell viability, antibiotic resistance and nucleic acid transfer. Interestingly, EVs are also hypothesized to mediate communication between microorganisms and the host organism; however, the nature of the molecular “message” they convey remains to be elucidated [93].

The composition and metabolic capacity of the gut microbiota varies significantly between individuals, which directly influences the profile of postbiotics produced in situ. These differences arise from factors such as diet, genetics, environment, and antibiotic exposure, and may contribute to individual variability in disease susceptibility, including cancer. The metabolic interplay between nutrition and the gut microbiota results in the biotransformation of inactive dietary precursors into potent anticancer compounds, acting as mediators of a complex host–microbe signaling axis. These molecules act as chemical messengers that bridge the gap between the gut lumen and systemic tissues, including the mammary gland [94]. For instance, SCFAs and tryptophan-derived indoles function as ligands for specific host receptors (e.g., GPR43, AhR), triggering intracellular cascades that regulate immune homeostasis and suppress oncogenic pathways [95,96]. Furthermore, through the inhibition of histone deacetylases (HDACs), postbiotics exert epigenetic control over host gene expression, reinforcing the notion that the microbiota–host interaction is a dynamic mechanism of reciprocal regulation rather than a one-way process [90]. Overall, this signaling axis serves as a molecular link that translates microbial metabolism into systemic protection against breast cancer progression in aging and obese hosts.

Recent research has also revealed a strong association between alterations in the gut microbiome and breast cancer, suggesting that microbial imbalance may influence systemic inflammation, endocrine regulation, immune function, and the composition of the tumor microenvironment [97]. Emerging evidence now indicates that the gut microbiota can regulate systemic inflammation and immune responses and thus may influence the effectiveness of both conventional and immunotherapeutic strategies for the treatment of breast cancer [98]. The gut microbial community influences drug pharmacokinetics—such as absorption, metabolism, and clearance—thereby altering both the efficacy and toxicity of conventional agents (chemotherapy, endocrine therapy) and emerging immunotherapies [99].

A key advantage of postbiotics is that they avoid the risk of transferring antibiotic resistance genes and virulence factors. Unlike probiotics, postbiotics exert their effects independently of bacterial viability, providing greater stability, safety, and reproducibility for therapeutic use [100]. Economically, postbiotics offer several benefits, such as a longer shelf life, easier storage and transport, as well as reduced need for refrigeration compared to probiotics [101]. In the field of oncology, postbiotics have garnered considerable interest due to their potential anticancer properties [102]. Experimental studies have demonstrated that certain *Lactobacillus*-derived postbiotics can selectively inhibit tumor cell proliferation, induce apoptosis, and modulate signaling pathways associated with cancer progression [103,104]. Furthermore, the immunomodulatory activities of postbiotics significantly contribute to their therapeutic potential. By modulating cytokine production, enhancing epithelial barrier integrity, and regulating oxidative stress, postbiotics can strengthen systemic immune responses and mitigate treatment-related adverse effects [105].

Postbiotics have emerged as a promising group of bioactive molecules with significant anticancer potential. An exploratory investigation based on isolated metabolites or macromolecules enabled the selection of the most promising components with antineoplastic activity, targeting different cellular events (Table 1). Among them, short-chain fatty acids, particularly butyrate, have been extensively studied for their role in promoting apoptosis, inhibiting tumor cell proliferation, and modulating epigenetic regulation through histone deacetylase inhibition [106]. Bacteriocins, which are antimicrobial peptides produced by lactic acid bacteria, demonstrate selective cytotoxicity against cancer cells by disrupting cell membranes and triggering programmed cell death [107]. Additionally, exopolysaccharides (EPSs) derived from microbial fermentation exhibit immunomodulatory effects, enhancing the activity of natural killer cells and macrophages, and have been shown to suppress tumor growth by interfering with cell adhesion and metastasis processes [108].

Although the evaluation of the antineoplastic potential of probiotic metabolites focuses mainly on processes directly involved in cancer growth and resistance, a review of the molecular and cellular mechanisms influenced by postbiotics provides a broader perspective on their potential significance in combatting breast cancer, particularly in the context of age and obesity as factors that favor carcinogenesis.

## 4. Anti-Senescent and Anti-Obesity Potential of Postbiotics in Context of Breast Cancer Prevention and Treatment

**Figure 2:**
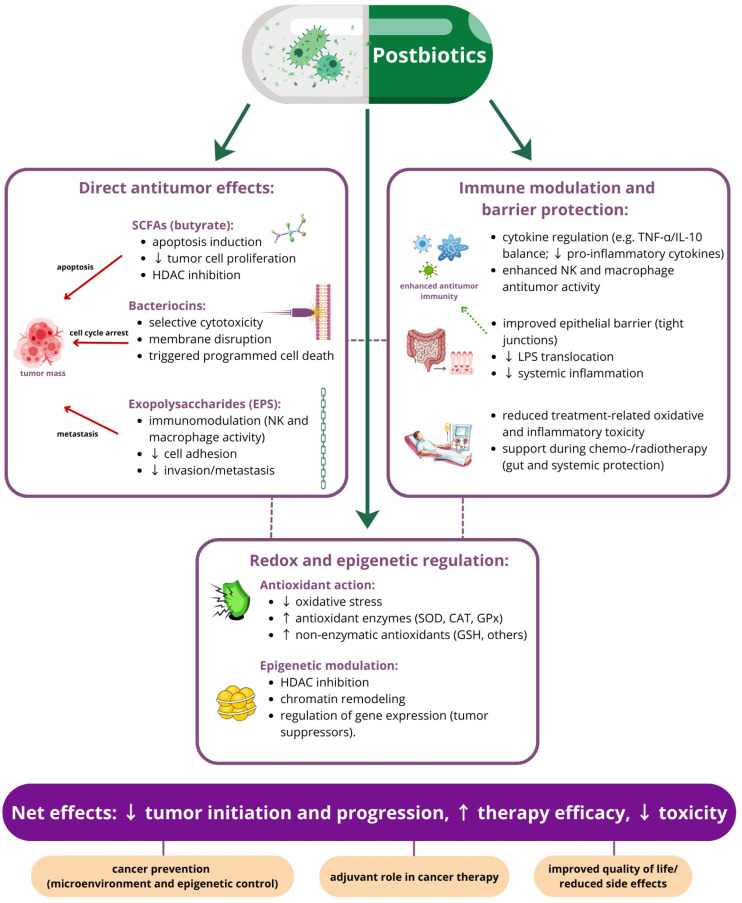
Figure illustrating the main mechanisms by which postbiotics may support cancer prevention and therapy. Postbiotic metabolites exert direct antitumor effects by inducing apoptosis, reducing tumor cell proliferation, disrupting cancer cell membranes, and limiting adhesion, invasion, and metastasis. They also promote immune modulation and barrier protection by regulating cytokines, enhancing NK and macrophage antitumor activity, strengthening the intestinal barrier, and reducing systemic inflammation and treatment-related toxicity. Finally, postbiotics contribute to redox and epigenetic regulation by lowering oxidative stress, increasing antioxidant defenses, and modulating gene expression via HDAC inhibition, together leading to reduced tumor initiation and progression, improved therapy efficacy, and lower toxicity. Green arrows represent the promotion of beneficial biological processes, while red arrows indicate the inhibition of tumor progression, specifically through apoptosis induction, cell cycle arrest, and metastasis suppression. CAT—catalase; GPx—glutathione peroxidase; GSH—glutathione; HDAC—histone deacetylases; LPS—lipopolysaccharides; SOD—superoxide dismutase; ↑—up; ↓—down.

The phenomena of aging and obesity is grounded in several common cellular and physiological events—including oxidative stress, chronic pro-inflammatory phenotype and enrichment in senescent cells—the factors creating a pro-carcinogenic microenvironment. Collectively, postbiotic metabolites act through complementary mechanisms, including modulation of oxidative stress, immune regulation, and direct tumor cell targeting, underscoring their potential to develop as novel, safe, and multifunctional agents in anticancer therapy (Figure 2).

### 4.1. Anti-Senescent Potential of Postbiotics

The emerging interest in the role of microbiota in human health has yielded results that suggest the versatile anti-aging potential of probiotics, or at least their ability to maintain good health at an older age. In the background of pro-health properties of ‘gerobiotics’, their antioxidant and anti-inflammatory activity have been found to possibly counteract perturbations in oxidative and inflammatory pathways promoting SASP, being the most detrimental effect of cellular senescence. The supportive role of probiotics can be executed by indirect influence via the immunological system and improvement of gut-barrier functionality, but also directly through secretory metabolites that traverse intestinal barriers and target distal organs and tissues, e.g., SCFAs, polyamines, and fermented polyphenols, that open avenues to unveil the pro-health benefits of postbiotics [115,116,117].

Recent reports show that gut dysbiosis, which is observed in postmenopausal women, may impact hormonal balance and indirectly influence metabolic health and cancer risk development, acting partially through changes in microbiota metabolites, such as the decline of beneficial SCFAs [118,119]. On the other hand, an increasing number of studies are showing the positive effects of probiotics and parabiotics, but also of postbiotic supplementation on hormonal balance, homeostasis maintenance and alleviating undesired symptoms in women around the menopause [23,118,120]. As microbiota plays a pivotal role in regulating estrogen levels, such an outcome may be the consequence of an increase in estrogen [121]. The ingestion of probiotics has been demonstrated to be positively associated with estradiol (E2) levels in premenopausal women; however, no such significant correlation was detected in postmenopausal women [122]. Moreover, probiotics have been demonstrated to augment the salutary effects of estriol and isoflavones when used as adjuncts to these treatments during the menopause transition [123]. However, there is limited evidence of a relationship between the estrobolome and breast cancer development. Further analysis is therefore essential to evaluate the potential benefits of probiotic supplementation for menopausal women, regarding the increased cancer risk that may result from a shift in microbiota function towards greater estrogen recycling [124,125].

Considering this literature gap, the findings indicating the promising potential of para- and postbiotics, as alternatives which can be useful in alleviating menopausal symptoms and maintaining overall homeostasis in middle-aged women, deserve more attention. Several recent reports on animal models, supported by further clinical investigation, demonstrate the ability of para- and postbiotics to protect bone loss, impact on the restoration of pelvic floor muscle function, improve inflammatory status and endocrine function, mitigate mild climacteric symptoms, and improve skin elasticity and moisture [126,127,128].

The versatile anti-aging effects resulting from postbiotic supplementation seem to be underpinned by their multifaceted bioactivity, including the antioxidant, anti-inflammatory, and anti-senescent potential, which counteracts molecular and cellular processes that favor aging.

The antioxidant properties of postbiotics are associated with a range of non-enzymatic compounds, including short-chain fatty acids and organic acids, glutathione, folic acid, phenolic metabolites, peptides, and extracellular polysaccharides. The postbiotics also contain enzymatic antioxidants, such as superoxide dismutase, catalase, glutathione, and peroxidase. The antioxidant potential of postbiotics has been validated by a variety of in vitro and in vivo assays; however, their effectiveness and mechanism of action appear to be contingent upon the specific probiotic strain and its metabolic output [129]. Antioxidant properties of postbiotics have been evaluated for single metabolites or enzymes, but also for mixtures of metabolic products obtained as LAB postculture supernatants or parabiotics applied as intracellular content or non-viable strains of different LAB, e.g., *L. fermentation*, *L. plantarum*, *L. rhamnosus*, *L. casei*, *L. acidophilus*, *L. paracasei* or *Bifidobacterium* species. To investigate the mechanisms of oxidative stress reduction at the cellular level, a variety of in vitro analyses has been performed, unveiling post- or parabiotics capacity to scavenge radicals, suppress the oxidation of cellular molecules (e.g., linoleic acid), and enhance the antioxidant capacity of enzymes (e.g., glutathione peroxidase) [130].

Several in vivo studies on animal models subjected to the induction of oxidative stress by chemical agents, dietary pattern or behavioral changes confirmed that postbiotics supplementation can enhance the body’s antioxidant defense system, improve serum biochemical profile, and mitigate the harmful effects of oxidative tissue damage via boosting the functionality of antioxidant enzymes, reducing the presence of oxidative and inflammatory stress markers (OSi; H_2_O_2_) and enhancing the production of antioxidant peptides [129,131,132]. Notably, probiotics and postbiotics also proved to be effective antioxidants in the case of aging mice. Measurements of oxidative stress indicators, levels of which increase with age, such as lipid oxide in propylene glycol (MDA), protein carbonyl after protein oxidation and mitochondrial 8OHdG content, demonstrated that a combination of pro- and postbiotics was effective in diminishing the negative effects of oxidative stress at the level comparable to resveratrol, or even better [133].

Antioxidant activity of postbiotics may underlie their anti-inflammatory action observed at cellular level in vitro and verified further by in vivo assays. In vitro studies conducted on macrophages, evaluating the postbiotic mixture derived from a variety of LAB strains or extracted macromolecules, such as exopolysaccharides, demonstrate that their anti-inflammatory action can be exerted via reducing cell phagocytosis and nitric oxide production, influencing the balance of pro- and anti-inflammatory cytokines (e.g., TNF-α/IL-10 ratio), favoring the latter. This phenomenon has been detected also in environmental stressor presence [134,135].

A comprehensive review of recent in vivo studies performed by Zdybel at al. demonstrates the multifaceted anti-inflammatory effects of post- and parabiotics exerted by modulating cytokine expression, influencing immune cell signaling pathways, regulating immune mechanisms such as the Th1/Th2 and Treg/Th17 equilibrium and strengthening epithelial barrier integrity [136]. Up to date, the majority of reports have been focused on postbiotics applications in inflammatory bowel diseases, showing their supportive and therapeutic implications exerted via capacity to elevate the key antioxidant enzymes and anti-inflammatory cytokines, as well as their ability to enhance mitochondrial function and promote biogenesis, thus boosting the defense mechanisms against oxidative damage and inflammation [44,137,138,139]. The results on animal models strengthened by the initial output of human studies/clinical trials indicate the potential use of post- and parabiotics as adjuvants supporting treatment of other diseases with an inflammatory background, such as autoimmune diseases or metabolic syndrome [136,140].

Oxy-inflammaging, a phenomenon triggered by oxidative stress and sustained by chronic immune disbalance, is currently considered one of the key factors underlying age-related and metabolic diseases. Importantly, gut microbiota dysbiosis is a hallmark of aging, associated with reduced microbial diversity, including SCFA-producing bacteria and increased inflammation. Intestinal microbiota disbalance contributes to several metabolic disturbances, but it has also been detected in oncological patients, suggesting its significance in cancer pathogenesis. Dietary patterns strongly influence aging-related health outcomes, with poor diet triggering inflammation and oxidative stress, and consequently promoting aging acceleration.

On the other hand, several recent reports present probiotic bacteria as longevity promoters via their anti-inflammatory, antioxidant and anti-immuno-senescence effects and indicate the postbiotics, e.g., SCFAs or polyamines, as direct modulators of age-related cellular events [117,141]. An output of recent findings examining postbiotics bioactivity and their health-beneficial effects creates them a promising tool counteracting the abovementioned phenomenon.

Multifaceted anti-aging properties of postbiotics have been summarized by Hamidi et al. [142]. Their antioxidant and anti-inflammatory activity, targeted directly to molecular events, creates a background for their anti-senescent potential at the cellular level. Postbiotics can exhibit anti-senescence activity by inhibiting PI3K/AKT/mTOR, a central aging-related pathway, which the signaling activity contributes to senescence progression. Modulation of NF-κB inflammatory signaling via attenuation of NF-κB activation and inhibition of MAPK pro-inflammatory pathways is the main mechanism resulting in reducing the inflammatory response by postbiotics [143]. Kumar et al. demonstrate that exposure of preadipocytes to *L. fermentum*-derived postbiotics significantly attenuated phosphorylation of PI3K/AKT/mTOR pathway and alleviated senescence markers p53, p21WAF1, SA-β-gal, p38MAPK, iNOS, COX-2, ROS, NF-κB, and DNA damage response induced by external stressor H_2_O_2_ [144].

Microbial products can also modulate the host epigenome, influencing the inflammatory status or the expression/activity of genes important in the context of aging, such as sirtuins [145].

Oncotherapeutics are known to stimulate senescence, but the TIS phenomenon may paradoxically contribute to acquired cancer resistance, which is strengthened by SASP. It also contributes to bystander effects, reaching non-cancerous cells affected by the oxy-inflammatory microenvironment [46]. Thus, the involvement of agents with antioxidant and anti-inflammatory capabilities, such as postbiotics, may prevent cells from various stressors leading to disturbances in cellular processes and DNA damage, and they may also inhibit the self-reinforced mechanism of senescence promoted by SASP.

**Figure 3:**
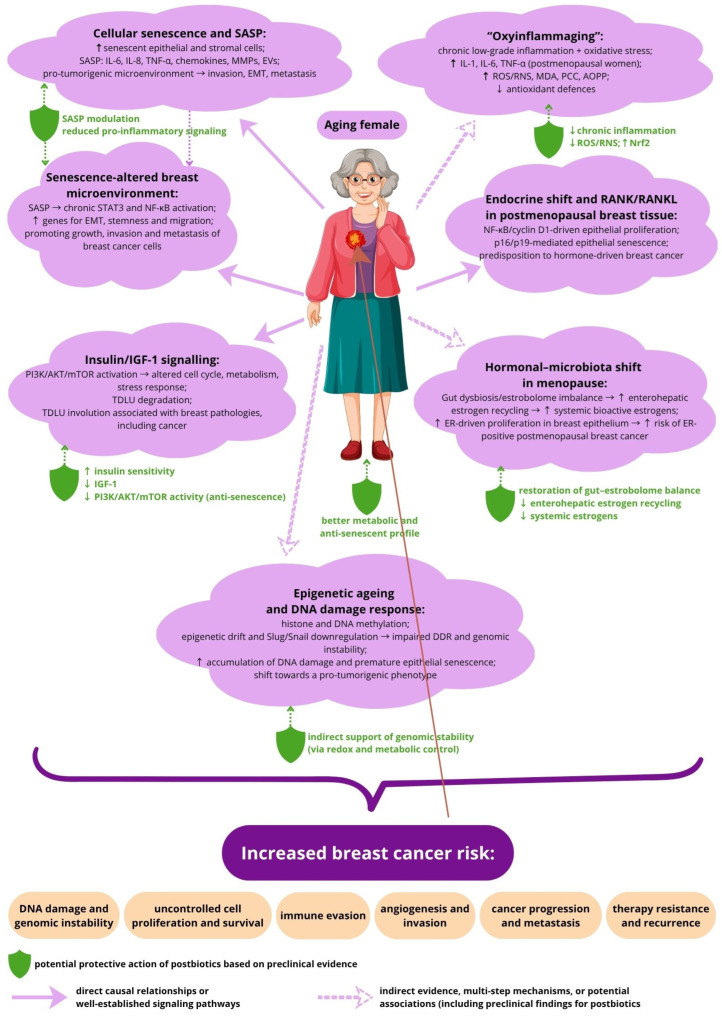
Figure summarizing aging-related mechanisms that increase breast cancer risk and indicating putative protective actions of postbiotics. Around the central ‘aging female’, seven hallmarks of aging are presented: cellular senescence/SASP, altered breast microenvironment, oxy-inflammaging, endocrine shift (RANK/RANKL), hormonal–microbiota shift, insulin/IGF-1 signaling, and epigenetic aging with impaired DNA damage response (DDR). Senescent cells and SASP factors contribute to a pro-tumorigenic microenvironment with chronic STAT3/NF-κB activation, EMT, and invasiveness, while oxy-inflammaging and endocrine alterations may further promote proliferation in postmenopausal tissue. The hormonal–microbiota shift represents estrobolome-driven disruption of gut–estrogen crosstalk, associated with increased estrogen recycling and ER-dependent proliferation. Altered insulin/IGF-1 signaling and epigenetic drift are linked to genomic instability. Green shields denote proposed postbiotic effects: modulation of SASP and inflammatory signaling, reduction in chronic inflammation and ROS/RNS with Nrf2 activation, improvement of insulin sensitivity with partial PI3K/AKT/mTOR down-regulation, restoration of gut–microbiota/estrobolome balance with more favorable estrogen homeostasis, and indirect support of genomic stability via redox and metabolic control. Solid arrows represent direct or well-established causal pathways, while dashed arrows indicate indirect mechanisms or associations requiring further clinical validation. AOPP—advanced oxidation protein products; DDR—DNA damage response; EMT—epithelial–mesenchymal transition; EVs—extracellular vesicles; MDA—malondialdehyde; MMPs—metalloproteinases; PCC—protein carbonyls; SASP—senescence-associated secretory phenotype; TDLU—terminal duct lobular units; ↑—up; ↓—down.

Apart from local microenvironmental cytoprotective action, postbiotics may positively influence biochemical and physiological processes important for healthy aging and minimizing the risk of age-related disease development, including cancer (Figure 3). Postbiotics can modulate gut microbiota composition, restore healthy microbial environment and metabolome. They can support gut-barrier protection by strengthening tight junction proteins (claudin, occludin, ZO-1) and reducing intestinal permeability common in aging, while improved gut-barrier function reduces systemic inflammation and metabolic dysfunction progressing further to disease development. Postbiotics can also impact metabolic pathways associated with longevity, including lipid regulation and glycemic control [142]. A versatile bioactivity of postbiotics considered in the context of their ant-aging potential makes them an interesting tool to undertake investigation aimed at diseases caused by inflammaging.

### 4.2. Anti-Obesity Activity of Postbiotics

Postbiotics have a multidimensional effect in counteracting obesity by modulating the gut microbiota, inflammation, adipogenesis, and energy homeostasis. Scientific research proves that probiotic metabolites exert potent anti-adipogenic effects by reducing adipocyte size, lipid content, and differentiation markers. They are capable of inhibiting the differentiation of preadipocytes into mature adipocytes through the downregulation of master adipogenic transcription factors and promoting lipolysis in established fat cells, thereby reducing adipose tissue expansion and improving metabolic health [146]. Postbiotics, particularly SCFAs, strengthen the integrity of the intestinal barrier, reduce endotoxemia, and promote beneficial bacteria (e.g., *Bifidobacterium*, *Akkermansia*), alleviating dysbiosis-associated obesity [147]. In addition, SCFAs affect the gut–brain axis by regulating appetite and satiety hormones, leading to reduced energy intake and improved metabolism. Acetate, propionate, and butyrate activate GPR41 and GPR43 receptors on enteroendocrine L cells in colon, which increases secretion of glucagon-like peptide-1 (GLP-1) and peptide YY, thereby reducing appetite and delaying gastric emptying [39]. Simultaneously, SCFAs lower ghrelin (“hunger hormone”) and improve leptin sensitivity, signaling satiety to the brain [148]. Moreover, probiotic byproducts can induce beiging of white adipose tissue, upregulating uncoupling protein 1 (UCP1) and converting white adipocytes into beige adipocytes with enhanced mitochondrial content and thermogenic capacity, thereby dissipating stored fat as heat and increasing energy expenditure [149].

Studies have also shown that, muramyl dipeptide (MDP), a postbiotic derived from bacterial cell walls, reduced adipose tissue inflammation and glucose intolerance in obese mice without causing weight loss or altering the composition of the microbiome [150]. In turn, surface layer proteins (SLPs) isolated from probiotic lactic acid bacteria (e.g., from kefir and kimchi) exhibit strong anti-inflammatory effects in immune cells and inhibit obesity in mouse models on a high-fat diet by improving inflammation, adipogenesis, and insulin resistance. In adipocytes, SLPs reduce lipid accumulation and induce apoptosis, highlighting their preventive potential in obesity-related metabolic disorders [149].

There are also in vivo studies on the effect of exopolysaccharides and bacteriocins from probiotic bacteria as anti-obesity agents. In mice fed a high-fat diet, EPS supplementation reduced body fat, lowered TAG levels in the liver and serum, and reduced inflammation [151]. In a similar study, EPS inhibited the differentiation of immature cells into mature adipocytes by upregulating AMPK signaling pathways and downregulating the expression of adiponectin and adipogenesis markers. Additionally, EPS exhibited anti-adipogenic action in the early stages of adipocyte differentiation [152]. In turn, *Lactobacillus*-derived bacteriocins in mice fed a high-fat diet reduced body weight and food intake, and in another study inhibited weight gain by reducing adipocyte size [153,154].

The effectiveness of postbiotics in alleviating obesity has also been demonstrated in some human studies. The administration of inulin-propionate ester to individuals in the BMI range of 25–40 kg/m^2^ significantly increased postprandial PYY and GLP-1 secretion and reduced calorie intake. In comparison with the control group, long-term administration of the supplement resulted in a significant reduction in weight gain and abdominal fat distribution, as well as a reduction in the amount of lipids in liver cells [155]. In another study, administration of acetic acid in the distal colon of obese patients resulted in increased fat oxidation and PYY concentrations compared to the placebo group [156]. Overall, a meta-analysis by Li et al. comprising 25 randomized controlled trials showed that postbiotic supplementation significantly reduces insulin, triglycerides, waist circumference, and CRP levels, without significant changes in body weight or BMI. The effects were stronger in younger participants (<50 years), with longer interventions (>8 weeks), and in bacterial-based or SCFA (butyrate) formulations [157].

Postbiotic supplementation may also potentially benefit the health of postmenopausal women, who often experience both dysbiosis and excess weight. However, current research is limited to evaluating the effects of probiotic bacteria. In a clinical trial in which obese postmenopausal women received multi-strain probiotics for 12 weeks, reductions in waist circumference, LPS, glucose, insulin, and HOMA-IR were observed, as well as improvements in lipid profile and inflammation [158].

Obesity significantly complicates breast cancer treatment in women, increasing risks of adverse effects, treatment toxicity, and poorer oncological outcomes. In obese patients, chemotherapy is linked to higher toxicity, reduced pathological complete response (pCR), and lower recurrence-free survival, often due to dose underestimation. Surgery carries a twofold increased risk of wound infections, thrombosis, and reconstruction failures, while radiotherapy elevates skin damage. Additionally, obesity attenuates the efficacy of tamoxifen and aromatase inhibitors via elevated endogenous estrogens from adipose tissue [159,160].

**Figure 4:**
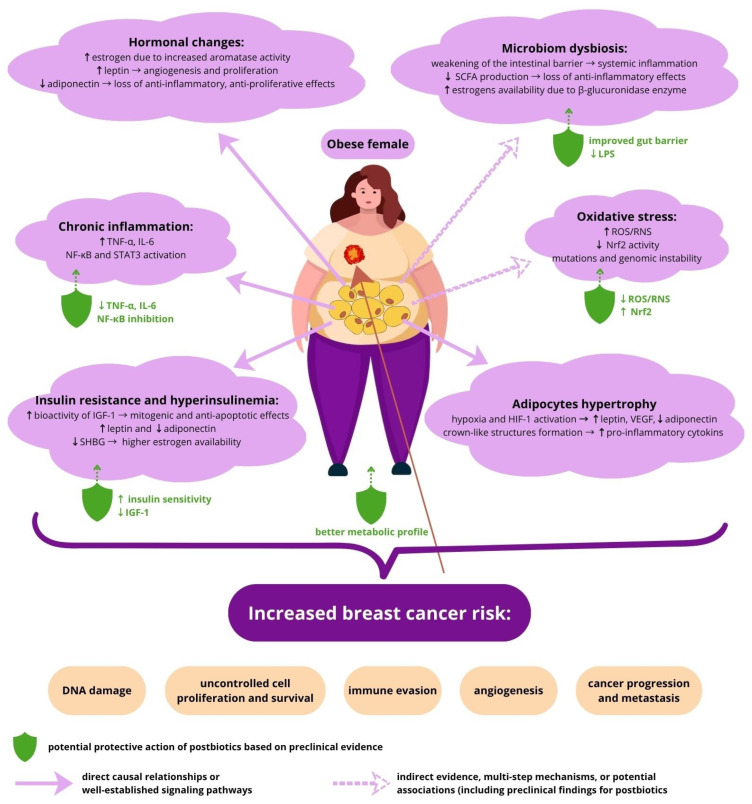
Figure summarizing major obesity-related mechanisms that enhance breast cancer risk and indicating putative protective actions of postbiotics. Surrounding panels depict six main pathways: hormonal alterations, chronic inflammation, insulin resistance and hyperinsulinemia, adipocyte hypertrophy, microbiome dysbiosis, and oxidative stress. Combined, these processes promote DNA damage, uncontrolled cell proliferation and survival, evasion of the immune response, angiogenesis, and ultimately breast cancer progression and metastasis. Green shield icons indicate sites at which postbiotics may counteract these mechanisms by improving gut-barrier integrity and lowering LPS, inhibiting NF-κB-driven inflammation, antioxidant activity, and ameliorating metabolic disturbances through improved insulin sensitivity and reduced IGF-1 levels, thereby partially counteracting obesity-driven breast carcinogenesis. Solid arrows represent direct or well-established causal pathways, while dashed arrows indicate indirect mechanisms or associations requiring further clinical validation. IGF-1—insulin-like growth factor 1; LPS—lipopolysaccharides; SHBG—sex hormone-binding globulin; VEGF—vascular endothelial growth factor; ↑—up; ↓—down.

Numerous scientific articles point to the significant potential of postbiotics in prevention and supportive therapies for obese women with breast cancer, which is based on reducing inflammation, rebuilding the microbiome, antioxidant activity and improving the metabolic profile (Figure 4) [161].

Biosynthetic metabolites produced by probiotic bacteria downregulate the production of pro-inflammatory cytokines (TNF-α, IL-6), which counteracts chronic inflammation typical of obesity and conducive to breast carcinogenesis. Postbiotics play a key role in restoring healthy gut microflora and strengthening the integrity of the intestinal barrier, thereby preventing the translocation of endotoxins, which stimulate a systemic inflammatory response. Exopolysaccharides and SCFA inhibit tumor cell proliferation and have antioxidant properties, protecting against damage resulting from chronic oxidative stress and genetic mutations [162]. Moreover, postbiotics have a positive influence on the body’s energy metabolism, supporting lipids and glucose homeostasis. In obese individuals, they reduce adipogenesis and improve fat burning, which translates into a decrease in risk factors for breast cancer [163].

A promising output of preclinical studies supports further investigations targeted to evaluating the utility of postbiotics in prophylaxis and treatment of dysfunctions initiated by oxidative stress and pathological states with an inflammatory background, including metabolic and age-related diseases (Table 2).

## 5. Multifaceted Potential of Postbiotics in Supportive Therapy of Breast Cancer and Alleviation of Side Effects

Postbiotics exert potent and selective inhibitory effects on malignant cells via several interconnecting molecular pathways. These mechanisms are often strain- and dose-specific, targeting the intrinsic vulnerabilities of cancer cells [161,165]. Epigenetic regulation, including DNA methylation and histone modifications, is a versatile mechanism through which postbiotics can alter the expression of target genes that are important for the pathogenesis and resistance of breast cancer [166]. Histone acetylation, which is controlled by histone acetyltransferases (HATs) and histone deacetylases (HDACs), is a significant modification in the context of postbiotic influence. In breast cancer, HDACs are often overexpressed, which ultimately results in the silencing of genes responsible for differentiation, apoptosis and cell cycle arrest [30,167]. SCFAs are the most well-documented postbiotic metabolites involved in the epigenetic reprogramming of breast cancer [168,169,170,171]. Butyrate and propionate act as natural, competitive inhibitors of Class I and Class II HDACs. The resulting hyperacetylation of histone tails has significant biological implications for breast cancer cells, including the reactivation of p21WAF1/CIP1, which is responsible for cell cycle arrest, and a shift in the Bax/Bcl-2 balance towards pro-apoptotic signaling [165,169,172].

Emerging evidence suggests that postbiotics can also significantly impact DNA methylation patterns, particularly by targeting the activity of DNA methyltransferases (DNMTs) [169]. Hypermethylation in the promoters of critical genes is found in 10-20% of sporadic breast cancers and is highly prevalent in the triple-negative subtype. The key genes with aberrant methylation status in breast cancer are: *BRCA1*, *RASSF1A*, *p16*, and *ESR1* (*ER*). By inhibition of DNMTs or promoter demethylation, postbiotics can restore expression of hypermethylated suppressors or estrogen-responsive genes [30,169,173]. Considering the proliferation and cell death as two major phenomena important for cancer therapy response and resistance, these processes have also been extensively investigated as targets for postbiotic action. Gosh et al. provided a comprehensive review pointing the cellular pathways influenced by postbiotics in breast cancer [161]. The anticancer potential of postbiotics at the cellular level can be manifested as an ability to induce intrinsic apoptotic pathways via Bcl-2/BAX modulation, mitochondrial cytochrome c release, and activation of caspase-9/-3, as well as the stimulation of extrinsic pathways via Toll-like receptors (TLRs), followed by caspase-8 activation. The proliferation of cancer cells can be influenced by postbiotics through the inhibition of Wnt/β-catenin signaling, which leads to the suppression of cyclin D1 and cell cycle arrest [161,162,165,174]. Apart from the direct orchestration of apoptosis and cell cycle control, postbiotics can also influence events in the tumor microenvironment important for cancer progression, growth and invasion. Postbiotics have the ability to downregulate matrix metalloproteinase (MMP) activity, minimizing the risk of metastasis. They also have the capability to reduce VEGF level, thus contributing to angiogenesis suppression [161,165].

One of the most significant functions of postbiotics in oncology is their ability to modulate the host immune system, thereby transforming an immunosuppressive tumor microenvironment into one that favors anti-tumor activity. This systemic reprogramming occurs through the direct interaction with immune cells and the indirect influence via gut-associated lymphoid tissue (GALT) [175]. Postbiotics have been shown to enhance the expansion and cytotoxic potential of natural killer (NK) cells, influence macrophage polarization and increase their phagocytic capacity. They also enhance the activity of CD8+ cytotoxic T cells and the antigenicity of tumor cells by upregulating MHC class I (HLA-I) on malignant cells. The interaction between postbiotics and the GALT via pattern-recognition receptors (PRRs) and G-protein-coupled receptors (GPCRs) is a major driver of systemic immune homeostasis, resulting in the upregulation of anti-inflammatory cytokines, such as IL-10, and the suppression of pro-inflammatory markers, like IL-6, IL-1, and TNF-α. Shifting the balance towards anti-inflammatory status is essential to prevent the pro-carcinogenic consequences of chronic low-grade inflammation [136,175].

These versatile mechanisms of postbiotic bioactivity underlie their antineoplastic potential and can provide complex support as adjuvants in oncology, including chemo-, immuno-, and endocrine therapy, as shown by preclinical evidence and a growing number of clinical trials with oncological patients.

### 5.1. Preclinical Evidence

The incorporation of postbiotics alongside cancer immunotherapies has demonstrated their potential tumor-inhibitory effects. By helping to reestablish a more diverse gut microbiota—commonly diminished in individuals with cancer—postbiotics may enhance the success of immune checkpoint blockade therapies [176]. These beneficial microbes can influence host immunity by stimulating key immune populations, including natural killer cells and T lymphocytes, which play essential roles in increasing effective antitumor responses. One of the study investigated by Aragon et al., implicate that in the mouse model, oral administration of *Lactobacillus casei* produced several notable antitumor outcomes, including a reduction in tumor growth rates, diminished tumor angiogenesis, and activation of CD8+ and CD4+T cells, which are essential components of the anticancer immune response [177].

Since probiotic-derived metabolites function as executors that target neoplastic cells and influence the cancer microenvironment and treatment responsiveness, an increasing number of studies have focused on evaluating the antineoplastic potential of postbiotics against breast cancer using in vitro models, with the results verified further through in vivo animal studies.

*Lactiplantibacillus plantarum* has been shown to exert direct anticancer effects on MCF-7 breast cancer cells by reducing cell viability, inducing nuclear condensation and fragmentation, and significantly increasing apoptotic cell populations, without a negative effect on normal cells. This effect was associated with an increase in pro-apoptotic BAX and cleaved PARP expression alongside a decrease in anti-apoptotic Bcl-2 levels, suggesting activation of internal apoptosis pathways [178]. Complementary findings indicate that postbiotics derived from *L. plantarum* and *L. rhamnosus* enhance the antineoplastic action of tamoxifen, a standard drug in breast cancer therapy, as well as a novel candidate drug (aziridine–hydrazide hydrazone derivative). In MCF-7 cells, the addition of LAB postbiotics not only reduced viability and promoted apoptosis but also shifted cells toward late apoptotic stages when combined with these drugs, producing stronger inhibitory effects on proliferation compared to chemotherapy alone. Cell-free supernatants also influenced the cell cycle, as evidenced by a shift toward the S phase. While postbiotics alone was capable of inducing apoptosis, primarily placing cells in the early stage of this process, the combination therapy significantly accelerated this effect, promoting progression to the late apoptotic phase [179]. Similarly, Dameshghian et al. demonstrated that postbiotics from *L. brevis* and *L. casei* selectively inhibited breast cancer cell proliferation and triggered apoptotic pathways, further confirming the broad-spectrum anticancer potential of LAB-derived metabolites across different strains [180]. Another study demonstrated that oral supplementation with *Lactobacillus acidophilus* in mice bearing 4T1 breast tumors significantly suppressed tumor progression. This treatment promoted a shift in cytokine secretion toward a Th1-dominant profile, a response associated with enhanced antitumor immunity [181]. Studies have also investigated the relationship between fermented dairy products, a natural source of postbiotics, and cancer. Zamberi et al. showed that in a BALB/c mouse group fed orally with kefir water, a significant reduction in tumor size and weight, along with a substantial increase in helper T cells and cytotoxic T cells, was observed in the kefir water-treated group [182].

Selective cytotoxicity of postbiotics against cancer cells are exerted mainly by influencing death- and viability-related pathways to inhibit cell proliferation and promote apoptosis, an activity which can also enhance the chemotherapy and sensitize resistant cells to treatment. Such effects have been detected also in breast cancer studies [161,183].

Apart from standard treatment approaches, novel therapeutic strategies have been explored that target pro-tumorigenic molecular pathways abolished in breast cancer [2]. Postbiotics, which possess versatile anticancer properties, can influence pathways that are commonly altered in neoplasms, providing potential support also for those precisive treatment approaches [143].

Constitutive activation of the PI3K/AKT/mTOR signaling pathways, which is detected in around 50% of ER-positive/HER2-negative breast cancers, has been postulated as a contributor to resistance to endocrine-based treatment. Therefore, searching for inhibitors that influence these pathways is considered a novel therapeutic strategy [2,184]. The PI3K/AKT/mTOR pathways are commonly indicated as targets influenced by postbiotics, and there is evidence that some LAB-derived postbiotics can prevent the activation of the PI3K/AKT/mTOR pathway, initially triggered by external stressors, subsequently alleviating inflammation and senescence phenomena [32,185].

NF-κB signaling, which contributes to tumor growth, metastasis, and responsiveness, could also be targeted in oncotherapy. The upregulation of the NF-κB pathway, a process that commonly accompanies cancer treatment, can paradoxically promote survival, proliferation and inflammation, ultimately enhancing breast cancer resistance [186]. A summary of reports presented by Jastrząb et al. suggests that postbiotics are molecules that attenuate NF-κB activity, which could make them potential inhibitors of pro-inflammatory signaling in treatment regimens. However, the authors also stated that the opposite effect has been observed, depending on the origin of the postbiotics used in various studies [143].

The classical treatment for HR-positive/HER2-negative breast cancer is based on tamoxifen and its derivatives—selective estrogen receptor modulators (SERMs). Endocrine therapy for advanced HR-positive breast cancer combines CDK4/6 inhibitors and aromatase inhibitors to block the cell cycle of cancer cells and decrease the level of estrogen, which is a proliferation trigger [2]. Postbiotics derived from LAB species have anti-proliferative potential against breast cancer cells, influencing cell cycle, and can boost the effect of tamoxifen in vitro. Moreover, the application of tamoxifen in vivo increases the number of *Lactobacillus* species in the mammary glands of mice, which subsequently reduces tumor formation [187].

The other effective options of supportive breast cancer therapy are immune checkpoint inhibitors (ICIs) [2]. Ferrari et al. demonstrate that postbiotics can sensitize cancer cells to treatment with immune checkpoint inhibitors by upregulating surface HLA class I expression, boosting the immunotherapy response [188].

Moreover, microbial metabolites, such as SCFAs, ellagic acids, and urolithins, have been shown to influence epigenetic events, including inhibition of HDAC and HAT activity, modulation of DNA methylation and histone modification, subsequently exerting anticancer effects [145].

Vrzácková et al. postulate that the anticancer effect of postbiotics, especially in early stages of carcinogenesis, is a consequence of the induction of cell response to metabolic and oxidative stress. The enhanced stress signaling protects normal tissues against malignancy by suppressing cell proliferation, mutagenesis and tissue inflammation. On the other hand, cancer cells usually exhibit internal ROS overproduction and enhanced stress signaling, triggering the progression of neoplasm due to its pro-survival effect. Authors suggest that postbiotics can overwhelm cancer cell’s adaptive stress response, even in the established malignant tumors, with a final anti-proliferative, pro-apoptotic and anti-metastatic effect [189].

Kim et al. suggest that postbiotics can act as external metabolic stressors, disrupting abnormal signaling pathways that sustain tumor growth. Notably, they exert a dual therapeutic effect: on one hand, they suppress tumor progression by attenuating oncogenic stress responses; on the other, they amplify oxidative and energetic strain selectively within malignant cells. This combination leads to the initiation of programmed cell death [190].

Considering the possible health benefits from postbiotics in oncotherapy, it is worth emphasizing that their anti-neoplastic potential is targeted to cancerous cells, but also the cytoprotective activity, which can be helpful in alleviating treatment side effects.

Current cancer therapies in breast cancer rely on conventional drugs that target malignant cells but also damage healthy cells, which can contribute to treatment resistance and make related therapies more difficult [191]. Such approaches often cause side effects, a frequent consequence of chemotherapy and radiotherapy, which diminish patients’ quality of life.

Experiments on animal models subjected to chemotherapy regimens alongside postbiotics produced promising results, particularly with regard to counteracting the inflammatory state induced by treatment. Oral administration of postbiotics exerts protective effects against cisplatin-induced renal damage in rats. Supernatants from probiotic bacteria, serving as postbiotic sources, inhibited the increase in IL-8 and TNF-α levels and did not induce inflammation in the kidneys. Additionally, in a group with cisplatin-induced nephrotoxicity, a reduction in kidney oxidative stress and DNA damage was observed. These findings suggest that bacterial metabolites may serve as adjunctive agents in anticancer therapy to mitigate treatment-related side effects. Also, Aragon et al. demonstrated that the administration of milk fermented by *Lactobacillus casei* delayed the tumor growth and increased mice survival. This outcome was associated with reduced tumor angiogenesis and alterations in the cytokine profile within the blood serum. Lower levels of IL-6 were linked to the preventive effects of this fermented milk [192]. Another major complication following chemotherapy is cardiomyopathy. Abu-Elsaad NM demonstrated that a probiotic diet containing yogurt with *Lactobacillus acidophilus*, green tea, and carrots reduced levels of TNF-α and IL-6 in rats after doxorubicin treatment. The tested diet also enhanced cardiac function and parameters by reducing serum levels of Angiotensin II and reduced ROS production [193].

Promising preclinical evidence on in vitro and animal models resulted in subsequent steps of investigation in patients with oncological diseases subjected to interventions with postbiotics per se or fermented foods, being a natural source of probiotic metabolites.

### 5.2. Current Clinical Evidence in Oncology and Translational Gaps

According to the available evidence, human studies investigating postbiotics in breast cancer remain limited. The presented clinical trials primarily focus on the safety of postbiotics and their potential therapeutic effects in reducing chemotherapy-associated side effects. However, the current advancement of the investigations has not yet provided conclusive results (Table 3).

Most completed clinical trials have focused on the use of probiotics or synbiotics, which can subsequently influence the composition of microbiota byproducts within organisms. In one clinical trial involving women with breast cancer undergoing chemotherapy, oral administration of β-glucan derived from *Saccharomyces cerevisiae* combined with *Lactobacillus rhamnosus* GG resulted in a slight increase in IL-12 levels and a significant reduction in serum IL-4 levels in the intervention group, suggesting potential immunomodulatory benefits [194]. Also, synbiotics supplementation may reduce treatment-related adverse effects during breast cancer therapy. Khazaei et al. reported a significant reduction in the severity of chemotherapy-associated complications, including abnormal bowel movements and fatigue after synbiotics supplementation (capsules containing 12 safe probiotic strains and fructooligosaccharides as prebiotic) in the synbiotic group compared with the placebo group [195]. Another interesting approach was presented by Raji Lahiji et al., who demonstrated that synbiotic intake significantly increased adiponectin levels and reduced TNF-α concentrations among 76 overweight or obese postmenopausal women with a history of hormone receptor–positive breast cancer [196].

Although the output of clinical trials implementing postbiotic interventions is still modest, the range of results yielded from in vitro models, through animal pre-clinical studies, demonstrates the ability of postbiotics to mitigate cancer treatment side effects, enhance immune response to cytotoxic therapy, and provide neuroprotective and anti-inflammatory benefits, potentially improving comfort and outcomes in oncological patients, including individuals with breast cancer. However, there is a clear need for more well-designed in vivo studies involving human participants to validate these beneficial effects and to confirm their safety, efficacy, and clinical relevance.

## 6. Conclusions—Perspectives and Limitations

According to current evidence, postbiotics exhibit pleiotropic pro-health effects, including antineoplastic, antioxidant, anti-inflammatory, and immunomodulatory properties. They effectively connect conventional probiotics with emerging next-generation therapies, providing notable advantages over widely used probiotic formulations, including a lower risk of promoting antibiotic resistance, improved ease of processing and greater stability during storage.

Considering that age and obesity are two major factors that contribute to the pathogenesis of breast cancer—both of which are underpinned by oxidative stress and inflammation, thereby boosting tumor progression—postbiotics could be beneficial for cancer prophylaxis. The dual effect of postbiotics, which selectively target cancer cells while acting as a protective agent on normal cells, makes them also a promising supportive option in oncotherapy. The multifaceted potential of postbiotics, which is rooted in their antioxidant, anti-inflammatory, and anti-senescent properties, means they could also counteract the treatment induced undesired effects, including the acquired resistance of cancer cells commonly leading to cancer recurrence.

The pathogenesis of breast cancer is related to disequilibrium in estrogen exposure, with the menopausal transition indicated as a turning point in hormonal balance and homeostasis. Therefore, postbiotic supplementation seems to be a safer adjunctive oncotherapy option than probiotic implementation, which could potentially boost estrobolome activity. However, further research is needed to clearly elucidate the interrelation of microbiota as estrogen-level modifiers and breast cancer risk development. Furthermore, the multifaceted bioactivity of postbiotics can prove advantageous in maintaining metabolic health and energy balance in a postmenopausal period of estrogen decline. In addition, it can act as a safeguard against age-related ailments, including an increased propensity for weight gain.

Direct clinical trials are necessary to evaluate the role of postbiotics in the treatment of breast cancer, especially in obese postmenopausal women, in whom intestinal microflora dysbiosis exacerbates inflammation and endotoxemia, worsening oncological prognosis. It is important to establish optimal doses and forms of postbiotics (short-chain fatty acids, surface proteins, extracellular vesicles) and their safety in the context of aging, and interactions with chemotherapy or hormone therapy. Postbiotics could act as adjuvants, improving chemotherapy tolerance by strengthening the intestinal barrier, reducing toxicity and modulating the immune response, whereas in obesity, they would support visceral tissue reduction, white adipose tissue beiging and appetite control.

However despite growing evidence supporting the therapeutic potential of postbiotics in cancers, several limitations and challenges must be addressed before their clinical applications. One major constraint is the substantial interindividual variability in gut microbiome composition, influenced by factors such as diet, genetics, environment, and medication use, which can lead to heterogeneous responses to postbiotic interventions. Additionally, the lack of standardization in postbiotic preparations—including differences in microbial strains, culture conditions, metabolite composition, and dosing—poses significant challenges for reproducibility and comparability across clinical studies. This variability complicates the identification of specific bioactive components responsible for anticancer effects. Addressing these challenges will be essential to unlock the full therapeutic potential of postbiotics and enable their translation into industrial practice.

## Figures and Tables

**Table 1:** Postbiotic compounds, their microbial sources, molecular targets, and reported anticancer mechanisms.

Postbiotic Compound	Microbial Sources	Molecular Targets	Anticancer Mechanism
Short fatty acids (e.g., butyrate, propionate, acetate)	*L. plantarum* *L. acidophilus*	HDAC, AMPK	Inhibition of histone deacetylases (HDACs), which leads to changes in gene expression that promote cell cycle arrest, differentiation, and apoptosis in cancer cells [109,110]
Bacteriocins (nisin)	*Lactococcus* *Lactis*	Cell membrane	Disrupting cell membranes and triggering programmed cell death in cancer cells [111]
Enzymes (catalase)	*L. plantarum * *L. acidophilus*	Reactive oxygen species (ROS)	Enhancing antioxidant capacity by reducing H_2_O_2_ levels and mitigating ROS induced intestinal damage [112]
Exopolysaccharides	*L. plantarum * *L. crispatus*	Immune receptors	Activation of immune cells and Toll-like receptor signaling [113]
Peptidoglycans	*L. paracasei*	Immune receptors	Reduction in intestinal inflammation and maintenance gut homeostasis [114]

**Table 2:** Postbiotics used in clinical trials to treat disorders involving inflammation and oxidative stress [164].

Trial No	Title	Interventions	Conditions
NCT06795425	A Randomized, Double-Blind Study to Assess the Effect of a Postbiotic on Oxidative Stress and Exercise Performance	Dietary Supplement: Postbiotic active lifestyle blend	Oxidative stress
NCT03378765	Gut Microbiota, Short Chain Fatty Acids, and Adiposity Across The Epidemiological Transition	Behavioral: Diet and physical activity monitoring	Obesity, Diabetes
NCT06911073	A Study to Evaluate a Postbiotic in Supporting Weight Loss and Metabolic Health	Dietary Supplement: Postbiotic	Obesity and Overweight
NCT04879914	Analysis of Microbiota Changes Induced by Microencapsulated Sodium Butyrate in Patients With Inflammatory Bowel Disease (IBDMicro)	Dietary Supplement: Butyrate	Inflammatory bowel diseases Irritable Bowel Syndrome
NCT05420805	Protective Role of Pre-/Post-biotics on Gut Inflammation, Dysbiosis, and Life Quality in Rett Syndrome (Biotics_RTT)	Dietary Supplement: ALAC, inulin, FOS, and sodium butyrate	Rett Syndrome, Dysbiosis, Epilepsy

**Table 3:** Clinical interventions with postbiotics in oncological patients [164].

Trial No	Title	Interventions	Conditions
NCT06097468	Nisin in Oral Cavity Squamous Cell Carcinoma (OCSCC)	Drug: NisinZ^®^ P	Oral Cavity Squamous Cell Carcinoma
NCT05736315	Use of a Fermented Dairy Beverage in Cervical Cancer Patients Undergoing Concurrent Chemoradiation Therapy	Other: Placebo dairy beverage	Cervical Cancer, Stage IIB
NCT06475807	Dietary Interventions in Cancer Patients Treated With Immune Checkpoint Inhibitors	Dietary Supplement: High-fermented food Dietary Supplement: High fiber supplementation	Cancer
NCT03177681	Fermented Milk Supplementation on Symptoms of Disease and Treatment in Patients With Multiple Myeloma	Dietary Supplement: Kefir	Plasma Cell Myeloma
NCT06517420	Effect of Kefir on Symptom Management and Quality of Life in Oncology	Other: kefir consumption	Breast Cancer Colorectal Cancer
NCT04795180	Pilot Randomized Evaluation of Butyrate Irrigation Before Ileostomy Closure on the Colonic Mucosa in Rectal Cancer Patients (BUTYCLO)	Drug: Butyrate irrigations trough the efferent limb of loop ileostomy	Rectal Cancer
NCT07268846	Use of Preoperative Postbiotic Supplementation in Colorectal Cancer Surgery	Dietary Supplement: Postbiotic	Cancer (Colon Cancer, Breast Cancer, Lymphoma, Chronic Lymphoma Leukemia, Multiple Myeloma)
NCT00006340	Ganciclovir Plus Arginine Butyrate in Treating Patients With Cancer or Lymphoproliferative Disorders Associated With the Epstein Barr Virus	Drug: Arginine butyrate Drug: ganciclovir	Leukemia Lymphoma Precancerous Condition
NCT00917826	Study of Arginine Butyrate and Ganciclovir/Valganciclovir in EBV(+) Lymphoid Malignancies	Drug: Arginine Butyrate Drug: Ganciclovir Drug: Valganciclovir	EBV Lymphomas Lympho-Proliferative Diseases

## Data Availability

No new data were created or analyzed in this study. Data sharing is not applicable to this article.
